# Predicting Depression in Patients With Knee Osteoarthritis Using Machine Learning: Model Development and Validation Study

**DOI:** 10.2196/36130

**Published:** 2022-09-13

**Authors:** Zuzanna Nowinka, M Abdulhadi Alagha, Khadija Mahmoud, Gareth G Jones

**Affiliations:** 1 MSk Lab Department of Surgery and Cancer, Faculty of Medicine Imperial College London London United Kingdom; 2 Data Science Institute London School of Economics and Political Science London United Kingdom

**Keywords:** knee osteoarthritis, depression, machine learning, predictive modeling

## Abstract

**Background:**

Knee osteoarthritis (OA) is the most common form of OA and a leading cause of disability worldwide. Chronic pain and functional loss secondary to knee OA put patients at risk of developing depression, which can also impair their treatment response. However, no tools exist to assist clinicians in identifying patients at risk. Machine learning (ML) predictive models may offer a solution. We investigated whether ML models could predict the development of depression in patients with knee OA and examined which features are the most predictive.

**Objective:**

The primary aim of this study was to develop and test an ML model to predict depression in patients with knee OA at 2 years and to validate the models using an external data set. The secondary aim was to identify the most important predictive features used by the ML algorithms.

**Methods:**

Osteoarthritis Initiative Study (OAI) data were used for model development and external validation was performed using Multicenter Osteoarthritis Study (MOST) data. Forty-two features were selected, which denoted routinely collected demographic and clinical data such as patient demographics, past medical history, knee OA history, baseline examination findings, and patient-reported outcome measures. Six different ML classification models were trained (logistic regression, least absolute shrinkage and selection operator [LASSO], ridge regression, decision tree, random forest, and gradient boosting machine). The primary outcome was to predict depression at 2 years following study enrollment. The presence of depression was defined using the Center for Epidemiological Studies Depression Scale. Model performance was evaluated using the area under the receiver operating characteristic curve (AUC) and F1 score. The most important features were extracted from the best-performing model on external validation.

**Results:**

A total of 5947 patients were included in this study, with 2969 in the training set, 742 in the test set, and 2236 in the external validation set. For the test set, the AUC ranged from 0.673 (95% CI 0.604-0.742) to 0.869 (95% CI 0.824-0.913), with an F1 score of 0.435 to 0.490. On external validation, the AUC varied from 0.720 (95% CI 0.685-0.755) to 0.876 (95% CI 0.853-0.899), with an F1 score of 0.456 to 0.563. LASSO modeling offered the highest predictive performance. Blood pressure, baseline depression score, knee pain and stiffness, and quality of life were the most predictive features.

**Conclusions:**

To our knowledge, this is the first study to apply ML classification models to predict depression in patients with knee OA. Our study showed that ML models can deliver a clinically acceptable level of performance (AUC>0.7) in predicting the development of depression using routinely available demographic and clinical data. Further work is required to address the class imbalance in the training data and to evaluate the clinical utility of the models in facilitating early intervention and improved outcomes.

## Introduction

Knee osteoarthritis (OA) is the most common form of OA and a leading cause of disability worldwide, with global prevalence estimated at 16% for individuals aged 15 years and over [1]. Knee OA is a chronic, progressive condition characterized by structural damage to the cartilage [2]. Knee OA results in chronic pain and impaired joint function, significantly limiting the activities of daily living [1,3]. Consequently, these patients experience a poorer health-related quality of life and are at higher risk of developing depression compared to the general population [4]. It has been estimated that up to 20% of patients with knee OA may be suffering from depression [3].

Several studies suggest that depression has an adverse impact on OA prognosis, quality of life, pain levels, as well as treatment effectiveness [5-7]. A longitudinal study conducted by Rathbun et al [8] found that depressive symptoms affected the physical functioning and pain severity of patients with knee OA. Another study showed that a persistently depressed mood significantly increases the severity of pain [9]. Additionally, a bidirectional relationship between pain and depression in patients with knee OA has been described, where concurrent depression increases pain perception and, reciprocally, higher pain levels may lead to a more depressed state [9-11]. It is therefore essential to recognize and address the vicious pain-depression cycle early.

Unsurprisingly, patients with knee OA and comorbid depression report lower coping ability, which translates into more frequent medical help-seeking and reduced satisfaction from treatment, including surgical interventions such as knee arthroplasty [3,10,12,13]. Ultimately, this accounts for a substantial rise in the health care cost burden [14,15]. Agarwal et al [16] estimated that the health care costs per year increase by US $4400 (US $13,684 vs US $9284) for every patient with concurrent OA and depression. The economic cost associated with knee OA is likely to rise in the upcoming years due to increasing life expectancy and thus the proportion of patients with knee OA [2]. With no curative treatment in sight, emphasis should be made on preventative and nonoperative strategies to manage the disease symptoms and reduce worsening factors such as depression [1,12].

Obtaining adequate mental health support should be of primary importance, as the presence of depressive symptoms is a significant predictor of worsening outcomes [17]. At the same time, appropriate therapy with antidepressants and counseling has been shown to significantly lower the perceived severity of pain [18]. However, less than half of all patients affected by knee OA and concurrent depression actively seek support or receive adequate treatment [19,20]. Unfortunately, poor mental health is frequently overlooked by clinicians, who focus primarily on the physical aspects of knee OA and so fail to recognize depression or its role in contributing to persisting knee symptoms [12,21]. Being able to predict which patients are at risk of experiencing depression would facilitate a targeted, preventative strategy against worsening outcomes such as pain and declining physical function [17].

Identifying patients with depression early would be helpful; however, no such tools currently exist. Although one previous study has tried to predict depression in this patient population, the model was based on conventional statistical methods, had low accuracy (﻿area under the receiver operating characteristic curve [AUC]=0.742, 95% CI 0.622-0.862), and lacked external validation [22]. This represents a significant gap in care. The solution may lie in machine learning (ML) models. The ability of ML algorithms to handle large data sets, and evaluate complex and nonlinear relationships between variables theoretically makes them better suited for predictive tasks than standard statistical methods [23,24]. To date, no previous study has attempted to build an ML prediction model to detect the development of depression in patients with knee OA.

The primary objective of this study was to apply ML models to predict depression in patients with knee OA, using routinely available clinical data. We hypothesized that ML models can deliver a clinically acceptable level of performance, defined as an AUC greater than 0.7. Our secondary objective was to identify the most important predictive features used by the ML algorithms to make this prediction.

## Methods

### Data Sources and Study Cohort

We used data from the Osteoarthritis Initiative (OAI) database for model development and data from the Multicenter Osteoarthritis Study (MOST) for external validation. Both are publicly available, prospective cohort studies investigating knee OA progression in the US population [25,26]. The OAI study included adults aged 45-79 years, enrolled between February 2004 and May 2006, and the MOST included adults aged 50-79 years, recruited in 2003.

We included patients who attended the baseline and 15-month/24-month follow-ups, with preexisting knee OA (defined as the presence of symptoms and radiographic evidence of OA) or at high risk of developing knee OA (symptoms of pain, stiffness, and swelling). Patients with a history of rheumatoid arthritis, missing data for the depression scale scores at either consultation, missing radiographic data, missing baseline examination findings, or missing patient-reported outcome measures were excluded.

### Ethics Considerations

No ethical approval was required for this study owing to the open access nature of the OAI and MOST databases.

### Prediction Outcome

Our primary outcome was the development of depression at 2 years following enrollment in the database. Depression was defined using the Center for Epidemiological Studies Depression Scale (CES-D), which is based on the Diagnostic and Statistical Manual of Mental Disorders, Fifth Edition formulation of depression, containing 20 questions evaluating the severity of psychosomatic symptoms [27]. The score ranges from 0 to 60, with higher values indicating greater symptom severity. A score of 16 points or more has previously been linked to clinical depression and as such was used in this study to dichotomize patients as either depressed or not depressed [27].

In the MOST, follow-up visits were scheduled at different time points compared with those used in the OAI study, and therefore CES-D scores captured during the 15-month visit were used for external validation.

### Variable Selection

Variable selection was guided by the literature and clinical relevance as judged by the senior author who is a specialist in the field. To facilitate external validation, equivalent variables had to be available in both the OAI and MOST data sets. In total, there were 2532 baseline variables in the OAI database and 1842 baseline variables in the MOST database; 70 and 66 variables were selected from the respective databases for model development. Variables included information on patient demographics, past medical history, knee OA history, baseline examination findings, and baseline patient-reported outcome measures.

Patient demographics included age, sex, ethnicity, BMI, marital status, living arrangements, current employment, education, and smoking status. Past medical history encompassed the history of heart attack, heart failure, stroke, asthma, chronic obstructive pulmonary disease, peptic ulcer disease, diabetes, kidney disease, and osteoporosis medication. Variables relating to knee OA history consisted of past knee injury, past knee surgery, steroid knee injections, analgesic medication for knee pain, as well as other arthritis medication. Baseline examination findings covered systolic and diastolic blood pressure, medial and lateral tibiofemoral, Kellgren-Lawrence grade, the 20-meter-walk test, the five-times-sit-to-stand test, and baseline CES-D score. Patient-reported outcome measures were the Western Ontario and McMaster Universities Osteoarthritis Index (WOMAC), Physical Activity Scale for the Elderly (PASE), and 12-item Short-Form Health Survey (SF-12).

### Data Preprocessing

#### Binning the Features

Smoking status was stratified according to smoking intensity into light (1-5 pack-year history of smoking), moderate (10-20 pack-years), or severe (>20 pack-years). BMI was grouped into underweight (BMI<18.5 kg/m^2^), normal weight (BMI 18.5-24.9 kg/m^2^), overweight (BMI 25-29.9 kg/m^2^), and obese (BMI>30 kg/m^2^), as defined by the World Health Organization [28]. Patients were categorized according to the American Heart Association Hypertension Guidelines to denote the stage of hypertension using variables for systolic and diastolic blood pressures [29]. Results of the five-times-sit-to-stand test were dichotomized, given that ≥10 seconds is the optimal cutoff for predicting the development of disability [30].

#### Feature Engineering

Feature engineering involves the combination of separate variables into a new, “engineered” feature, based on domain expertise and literature evidence. This action decreases the number of separate features and has been shown to improve model performance [31]. The “ethnicity” feature was created by merging variables describing race (*white, Black, Hispanic, other*). Variables assessing living arrangements were combined to denote whether the patient lived alone or with someone else. A feature for OA history was created by combining variables denoting the presence of other types of arthritis (*no other arthritis, one or more joints affected by OA, gout, OA and gout*). Variables denoting the use of analgesic medication for knee OA were assigned into a single feature, “analgesic medication” (*no pain relief, topical salicylates, nonsteroidal anti-inflammatory drugs or cyclooxygenase-2 inhibitors, opioid medication, combination of analgesic medication, other*). The “OA medication” feature was created by combining variables with information on OA treatment and supplements (*no medication or vitamin D supplements, bisphosphonates, estrogen/raloxifene, calcitonin/teriparatide, combination of OA medications*). The “arthritis medication” feature was created by merging five variables (*oral corticosteroids, supplements*). The final list of 42 features included in model training is summarized in Table 1.

**Table 1:** Summary of all features included in the model training.

Feature category	Features
Patient demographics	Age, sex, BMI, ethnicity, employment status, education status, living alone, marital status, smoking status
Past medical history and medication	Heart attack, heart failure, stroke, asthma, chronic obstructive pulmonary disease, peptic ulcer disease, diabetes, kidney disease, osteoporosis medication
Knee osteoarthritis history	Knee arthroscopy, knee meniscectomy, ligament repair, other knee surgery, arthritis of other joints, knee injury, steroid knee injections, analgesic medication for knee osteoarthritis, arthritis medication
Baseline examination findings	Blood pressure, 20-meter-walk test, five-stands-to-sit test, KLG^a,b^, CES-D^c^ baseline
Patient-reported outcome measures	WOMAC^a,d^ (Total, Pain score, Stiffness score); SF-12^e^ (Physical components, Mental health component); PASE^f^

^a^Separate feature for the right and left knee.

^b^KLG: Kellgren-Lawrence Grade.

^c^CES-D: Center for Epidemiological Studies Depression Scale.

^d^WOMAC: Western Ontario and McMaster Universities Osteoarthritis Index.

^e^SF-12: 12-item Short Form Health Survey.

^f^PASE: Physical Activity Scale for the Elderly.

#### Missing Values

Missing values in the OAI data set were addressed by coding them as “unknown” to match the MOST data set. Following this imputation, only patients with all observations completed were included for analysis.

### Model Development

#### Overview

**Figure 1:**
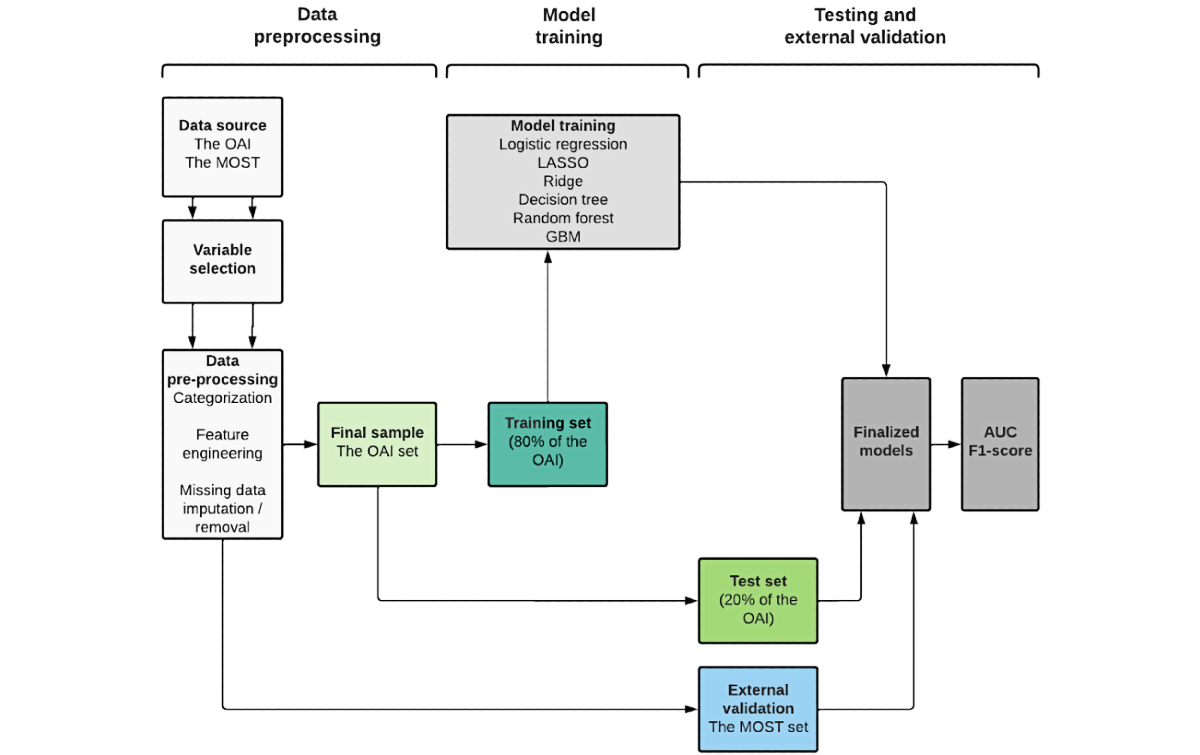
Flowchart summarizing the project timeline and steps of model development. AUC: area under the receiver operating characteristic curve; GBM: gradient boosting machine; LASSO: least absolute shrinkage and selection operator; MOST: Multicenter Osteoarthritis Study; OAI: Osteoarthritis Initiative.

Figure 1 summarizes the stages of data preprocessing and model development. The OAI data set was randomly divided into training (80% of observations) and test (20% of observations) sets using a computer algorithm, ensuring that each set included an equal proportion of patients with depression. Six common classification ML algorithms (logistic regression, least absolute shrinkage and selection operator [LASSO], ridge, decision tree, random forest, and gradient boosting machine [GBM]) were trained using the same set of 42 features. Classification models are a type of supervised ML where the algorithm calculates a probability of an observation belonging to the “positive” class based on the input data [32]. If the probability is above the threshold, the observation is labeled as “positive” (ie, depressed). The probability threshold is by default set to 0.5 but can be lowered when the cost of missing a “positive” case is high. Therefore, in this study, the threshold was set to 0.2 [33]. For each model, hyperparameter tuning was conducted until the performance on the training set was maximized. All models were developed using RStudio software (version 1.4.1106) [34].

#### Logistic Regression

Logistic regression is a statistical model that uses a logit function to predict the probability of an observation belonging to the positive class [35]. Logistic regression is well-suited for classification problems such as problems involving describing the risk of developing a disease or the risk of mortality. This model was implemented using the RStudio “stats” package [36].

#### LASSO and Ridge Regression

LASSO and ridge regression models are based on the logistic regression model [24,32,37]. In LASSO, the algorithm adds a “penalty” to each feature so that features are eliminated if not considered important for the prediction by the algorithm [37]. LASSO shrinks regression coefficients toward 0, and ultimately only top informative features are included. This results in a simpler and more easily interpretable model [37]. In ridge, the algorithm reduces less important features to close to zero but does not eliminate them [32]. In this way, all features are kept in the model, which is beneficial when all features need to be included [32]. LASSO and ridge models were developed using the “glmnet” package with optimal hyperparameters for both algorithms set as follows: nfolds=3, s=lambda.min [38].

#### Decision Tree and Random Forest

Decision tree is a simple, tree-shaped algorithm, in which each branch of the tree determines a possible decision or course of action [39]. The model was developed with no additional hyperparameters using the “rpart” package [40]. Random forest is an algorithm similar to the decision tree; it operates by building multiple, independently trained decision trees using random subsets of the data [41]. Subsequently, their predictions are combined into a single prediction outcome. Random forest of 500 trees with nodesize=100 and mtry=4 was developed using the “randomForest” package [42].

#### GBM Model

In GBM, multiple tree-based classifiers are trained to augment each other and to reduce the prediction error [43]. GBM differs from the random forest algorithm in that a new decision tree is trained with the aim to correct errors made by existing trees, rather than training them independently. This model was developed using the “gbm” package and optimum hyperparameters were ntrees=2000, cv.folds=3, interaction.depth=4, and shrinkage=0.1 [44].

### Performance Evaluation

The overall model performance was evaluated on the previously unseen OAI test set and externally validated using the MOST data set.

The primary model performance criterion was the AUC, and we considered an AUC greater than 0.7 to indicate clinically acceptable performance [45]. For each model, accuracy, precision, and recall are also reported. In addition, the F1 score, a weighed metric of precision and recall, was calculated according to the formula: F1=2×([precision×recall]/[precision+recall]). F1 score ranges from 0 (poor performance) to 1 (perfect performance).

While ML may provide a valuable predictive tool, the clinical implementation often raises concerns due to the model’s complexity, referred to as the “black-box” problem [46]. One way of improving model understanding is by extracting the most important predictive features. We therefore identified the most important predictive features from the best-performing model.

## Results

### Study Participants

**Figure 2:**
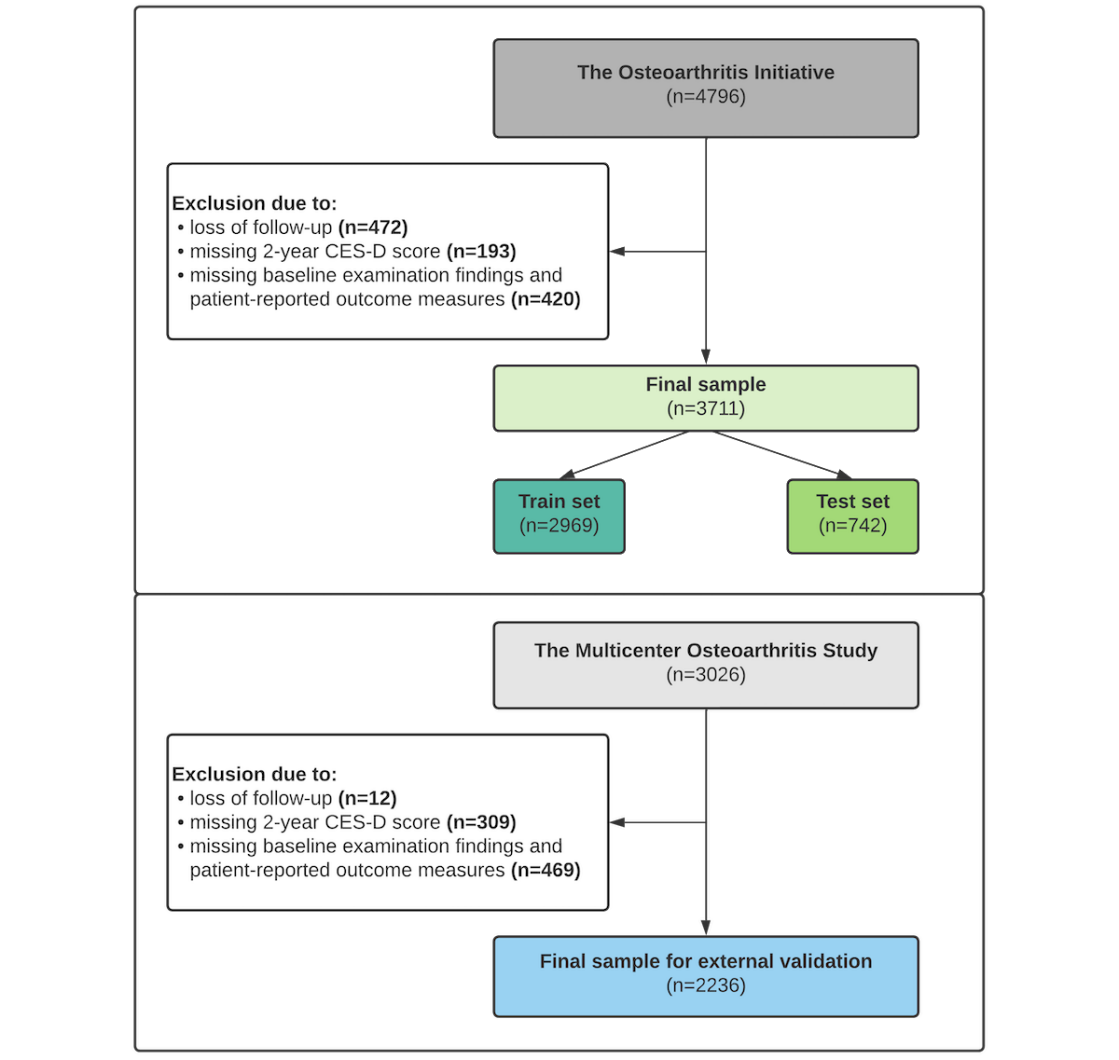
Summary of patient flow for both databases. CES-D: Center for Epidemiological Studies Depression Scale.

The initial OAI data set included 4796 patients (Figure 2). Following exclusion of 1085 patients, the final sample size encompassed 3711 patients. After splitting the sample, the training set included 2969 patients and the test set had 742 observations. In the MOST data set, 790 patients were excluded from the initial sample of 3026 cases and the final sample included 2236 patients.

Table 2 summarizes the key patient characteristics. The average age was 61.0 years for the OAI sample and 62.1 years for the MOST sample. In both data sets, the majority of patients were female and of white ethnicity. Less than half of the patients had hypertension stage 1 or higher. There were some differences between the OAI and MOST samples. First, the proportion of depressed patients at 2 years was higher in the MOST sample. The MOST population also had higher average WOMAC scores for both the right and left knees, and a greater proportion of patients using analgesic medication for knee OA.

**Table 2:** Key patient demographic and clinical data.

Characteristic			OAI^a^ (n=3711)		MOST^b^ (n=2236)
Age, mean (SD)			61.0 (9.1)		62.1 (8.1)
BMI, mean (SD)			28.4 (4.8)		30.4 (5.9)
Sex (female), n (%)			2149 (57.91)		1297 (58.01)
Ethnicity (white), n (%)			3082 (83.05)		1932 (86.40)
Blood pressure (hypertension stage≥1), n (%)			1847 (49.77)		1008 (45.08)
Other arthritis, n (%)			1454 (39.18)		1071 (47.90)
Analgesic medication for knee OA^c^ (any), n (%)			845 (22.77)		1804 (80.68)
**KLG^d^, n (%)**					
	Right knee, grade 1 or higher	2294 (61.82)		1180 (52.77)	
	Left knee, grade 1 or higher	2206 (59.44)		1264 (56.53)	
**WOMAC^e^-total, mean (SD)**					
	Right knee	10.7 (10.3)		18.6 (17.5)	
	Left knee	10.7 (10.4)		18.3 (17.5)	
Baseline CES-D^f^, mean (SD)			6.3 (6.0)		6.7 (6.2)
Depression at 2-year visit, n (%)			342 (9.22)		265 (11.85)

^a^OAI: Osteoarthritis Initiative.

^b^MOST: Multicenter Osteoarthritis Study.

^c^OA: osteoarthritis.

^d^KLG: Kellgren-Lawrence Grade.

^e^WOMAC: Western Ontario and McMaster Universities Osteoarthritis Index.

^f^CES-D: Center for Epidemiological Studies Depression Scale.

### Model Performance

**Figure 3:**
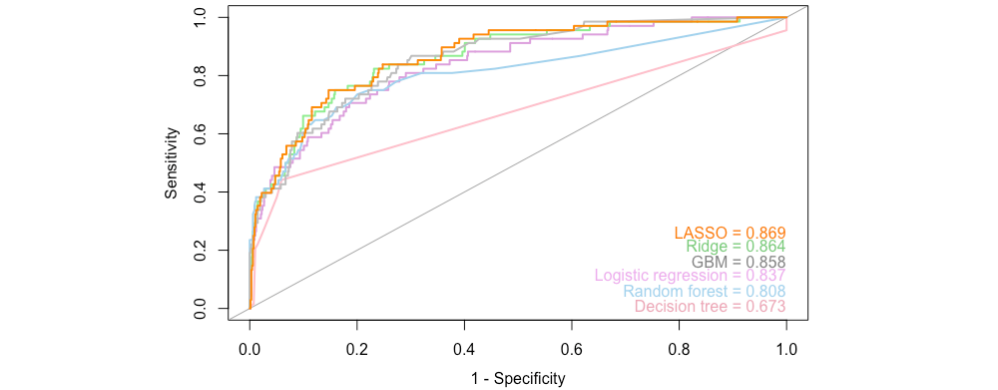
AUC plot of all models tested on the OAI test set (20% of the initial OAI data set). The test set was not used at any stage of model training. AUC: area under the receiver operating characteristic curve; GBM: gradient boosting machine; LASSO: least absolute shrinkage and selection operator; MOST: Multicenter Osteoarthritis Study; OAI: Osteoarthritis Initiative.

**Figure 4:**
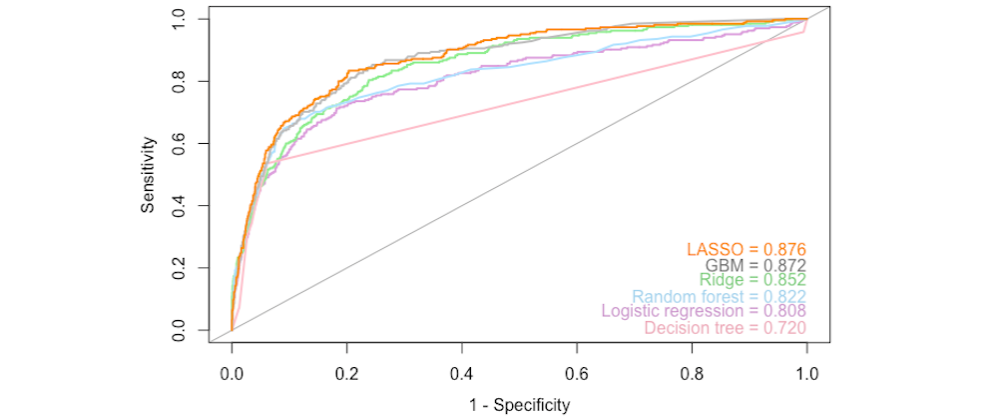
AUC plot of all models externally validated on the MOST data set. AUC: area under the receiver operating characteristic curve; GBM: gradient boosting machine; LASSO: least absolute shrinkage and selection operator; MOST: Multicenter Osteoarthritis Study; OAI: Osteoarthritis Initiative.

In total, six classification models were trained using all 42 features. The results for each model are summarized in Table 3. Figure 3 and Figure 4 present the AUC plots for the internal test set and the external validation set, respectively. The AUC ranged from 0.673 to 0.869 for the internal test set and from 0.720 to 0.876 for the external validation set. Except for the decision tree algorithm, all models yielded an AUC>0.7, suggesting clinically acceptable discrimination between depressed and nondepressed patients [45]. LASSO was the model with the highest AUC on both the internal test set and external validation set.

The accuracy, precision, recall, and F1 scores for the test and validation sets are summarized in Table 4 and Table 5, respectively. The accuracy on the OAI test set varied from 0.895 (decision tree) to 0.923 (random forest). The performance on this metric was lower for the MOST data set, ranging from 0.865 (GBM) to 0.895 (ridge). Despite high accuracy, the proportion of correctly classified positive cases was relatively low. For the internal test set, the F1 scores varied from 0.435 (decision tree) to 0.490 (LASSO), and from 0.456 (ridge) to 0.536 (LASSO) on external validation. LASSO had a consistently high performance for the AUC and F1 score in comparison to the other models, ranking first on both the internal test and external validation sets.

**Table 3:** Model performance for the internal test set and external validation set.

Rank^a^	Model	Test set (OAI^b^), AUC^c^ (95% CI)	External validation set (MOST^d^), AUC (95% CI)
1	LASSO^e^	0.869 (0.824-0.913)	0.876 (0.853-0.899)
2	GBM^f^	0.858 (0.813-0.903)	0.872 (0.849-0.895)
3	Ridge	0.864 (0.818-0.910)	0.852 (0.827-0.878)
4	Random forest	0.808 (0.741-0.874)	0.822 (0.790-0.853)
5	Logistic regression	0.837 (0.786-0.888)	0.808 (0.775-0.840)
6	Decision tree	0.673 (0.604-0.742)	0.720 (0.685-0.755)

^a^Models are ranked by their performance on the external validation data set.

^b^OAI: Osteoarthritis Initiative.

^c^AUC: area under the receiver operating characteristic curve.

^d^MOST: Multicenter Osteoarthritis Study.

^e^LASSO: least absolute shrinkage and selection operator.

^f^GBM: gradient boosting machine.

**Table 4:** Accuracy, precision, recall, and F1 scores for the test set, ranked by the F1 score.

Rank	Model	Accuracy	Precision	Recall	F1
1	LASSO^a^	0.902	0.467	0.515	0.490
2	Random forest	0.923	0.628	0.397	0.486
3	Logistic regression	0.906	0.485	0.485	0.485
4	GBM^b^	0.901	0.466	0.500	0.482
5	Decision tree	0.895	0.429	0.441	0.435
6	Ridge	0.908	0.500	0.426	0.460

^a^LASSO: least absolute shrinkage and selection operator.

^b^GBM: gradient boosting machine.

**Table 5:** Accuracy, precision, recall, and F1 scores for the validation set, ranked by the F1 score.

Rank	Model	Accuracy	Precision	Recall	F1
1	LASSO^a^	0.889	0.528	0.604	0.563
2	Decision tree	0.890	0.538	0.536	0.537
3	GBM^b^	0.865	0.453	0.657	0.536
4	Random forest	0.894	0.556	0.506	0.530
5	Logistic regression	0.886	0.344	0.698	0.461
6	Ridge	0.895	0.593	0.370	0.456

^a^LASSO: least absolute shrinkage and selection operator.

^b^GBM: gradient boosting machine.

### Most Important Predictive Features

The most important predictive features identified by LASSO were blood pressure, CES-D score at baseline, total WOMAC score for both knees, and mental and physical components of the SF-12 survey. Blood pressure had the highest coefficient (0.173), followed by the baseline CES-D score (0.126), WOMAC total for the right knee (0.004), and WOMAC total for the left knee (0.003). The mental and physical components of SF-12 had negative coefficients (–0.032 and –0.009, respectively).

## Discussion

### Principal Findings

The results of this study demonstrate that it is possible, with high accuracy, to predict depression in patients with knee OA using a variety of routinely collected data such as patient demographics, medical history, examination findings, and patient-reported outcome measures. The developed ML models achieved clinically relevant discrimination between depressed and nondepressed patients, with LASSO identified as the best-performing model, yielding an AUC of 0.876 (95% CI 0.853-0.899) on external validation. The accuracies for external validation were high, ranging from 0.865 (GBM) to 0.895 (ridge), meaning that between 86.5% and 89.5% of all patients were correctly classified. However, the F1 scores ranged from 0.456 (ridge) to 0.563 (LASSO). Low F1 scores despite high accuracy implies that the models can identify patients without depression more accurately than those with depression. This is likely due to class imbalance in the data set, which is a common problem in medical research that results in predictive modeling bias toward the majority [47].

While ML may provide a valuable predictive tool, the clinical implementation often raises concerns due to model complexity, often referred to as the “black-box” problem [46]. One way of improving model understanding is to extract the most important features [48]. In this study, blood pressure, the baseline CES-D, the total WOMAC, as well as mental and physical components for SF-12 were identified as being the most informative measures for prediction. Although this does not imply a statistically significant correlation between the features and the prediction outcome, it is reassuring that the input features identified by LASSO have previously been highlighted as factors associated with an increased risk of developing depression in patients with OA [8,9,49]. Surprisingly, blood pressure was identified as being the most informative factor for prediction. The presence of multiple comorbidities can further increase the risk of depression development in patients with knee OA, regardless of their pathophysiology [49]. Notably, the radiographic severity of OA was not highlighted as a predictive feature for depression development. This is consistent with previous research showing that depression and pain are independent from the extent of radiographic degenerative changes [50]. This known discrepancy between knee OA symptoms and radiographic severity highlights the complex nature of the disease and the need for more objective assessment tools. The association between depression, chronic conditions, and pain is complex. The temporality of the relationship between depression and pain has been poorly researched, but it appears that both factors potentiate each other, with higher pain severity increasing the persistence of depressed mood and the presence of pain increasing the incidence of depression [5,7,28,51,52]. This highlights the essential role of appropriate, interdisciplinary mental health support for patients with knee OA.

ML predictive models have an important role in augmenting clinical judgment, and when compared with standard predictions, they produce more accurate and less variable risk estimates [53]. The best-performing model in our study, LASSO, could be potentially used to aid in identification of patients at risk of future depression. Since the CES-D score has been designed as a screening tool, the patients identified as “positive” by our model would have to undergo further, more specialist mental health assessment. Depending on that outcome, the patients could be offered either a self-help aid, or potentially, a specialist referral. This would be more economical and time-efficient than assessing every patient attending with knee pain. However, further research is required since the implementation of predictive models is often difficult due to lack of clear clinical guidance on how to act upon the predicted outcome [54].

The advantage of our models lies in their simplicity as they rely on easily accessible clinical information. In addition, LASSO identified only 6 features to be crucial for prediction, making the model more practical. Blood pressure is routinely measured by primary health care practitioners, and WOMAC, SF-12, and CES-D scores are commonly used patient-reported outcome measures [55-57]. The aforementioned questionnaires are brief and require minimal training. Currently, there is no proven strategy to prevent or cure knee OA, and the therapy is focused on alleviating pain and addressing functional limitations [9]. Since depression is a potentially modifiable risk factor for worsening pain and function in knee OA, our prediction model could offer a targeted, preventative strategy. Diagnosing depression in patients with concurrent chronic pain conditions is challenging and having such information would facilitate discussions around the patient’s mental health, even at times when the patient is not yet aware of their symptoms. While further research is required to evaluate the practical aspects of the clinical application, the findings of our study represent an important step toward developing a potential diagnostic aid, addressing a significant gap in knee OA care.

### Comparison With Prior Work

To the best of our knowledge, this is the first study applying ML to predict depression in patients with knee OA. One previous study attempted to develop a prediction model based on logistic regression using conventional statistical methods [22]. Although the model achieved a clinically acceptable performance with an AUC of 0.742 (﻿95% CI 0.622-0.862), it was built using a small sample of patients and was not tested on an independent sample or externally validated [22].

Diagnosis of depression is challenging in clinical practice, and ML models have been previously applied to predict illness in different patient populations [58-62]. Clinically relevant predictive performance of common ML classification algorithms was shown in two studies predicting postpartum depression [58,59]. Cvetkovic [60] used a deep-learning approach to predict depression in breast cancer patients, achieving high internal accuracy. However, the study methodology was poorly reported, with information lacking on data preprocessing and model testing [60]. In another study, depression and anxiety in college students were estimated using GBM, with satisfactory performance yielding an AUC of 0.730 [61]. When applied to community-residing older adults, a logistic regression model achieved variable accuracy, ranging from 58.33% for severe depression to 90.44% for mild depression [62]. The variation in model performance achieved by these studies could be attributed to the use of different algorithms, different evaluation tools for detection of depressive symptoms, as well as the use of different predictive features.

### Strengths

Our study is strengthened by the use of a large patient cohort for model development, testing, and validation. The list of input features was carefully curated, with selection based on literature evidence, domain expertise, and data completeness. In addition, our predictive models were externally validated and performed well in an independent cohort, demonstrating their generalizability and potential for clinical application. Notably, LASSO identified only six features to be crucial for prediction, which showcases the simplicity of our method and the ease with which this tool could be used in a clinical setting.

### Limitations

Several limitations should be addressed in future research. First, the study sample used for model development might not be representative of a general population of patients with knee OA. The prevalence of depressed patients in the training set was 9.2%, which is much lower than the 20% rate previously suggested by the literature [63]. The OAI study excluded patients with end-stage OA, morbid obesity, or those with terminal diseases, whereas these factors are associated with an even higher risk of depression [25,49]. Second, both the OAI and the MOST data sets were based in the United States with patients from a predominantly white ethnic background [25,26]. Further validation of our prediction model in a more ethnically and socioeconomically diverse population would help to detect any potential discrimination. Third, due to differences in the OAI and MOST protocols, follow-up times differed by 15 months between the training and external validation sets. Nevertheless, the models were able to predict on the external data set with similar performance. Lastly, the presence of depression at 2 years was defined using the CES-D scale; although this tool has been validated for use in patients with chronic illness and OA, it is not considered a gold standard for the diagnosis of depression [27]. However, the CES-D questionnaire has the advantage of being brief, easy to understand, and requiring minimal training for the assessor [27].

### Conclusions

This is the first study to apply ML classification models to predict depression in patients with knee OA using routinely collected patient data. The LASSO model offered the highest quality of prediction, with an AUC of 0.876 (95% CI 0.853-0.899) on external validation. The advantages of our method include the use of a large patient cohort and routinely collected data, as well as external validation on an independent data set. This tool offers a potential opportunity to assess a patient’s risk of future depression, facilitating early intervention. Further research is required to establish where such a tool would fit within the care pathway, and while the harmful effects of depression on knee OA are well documented, it will be necessary to confirm that early detection and management of depression in this population leads to the expected improvement in outcomes.

## Abbreviations

AUC: area under the receiver operating characteristic curve
CES-D: Center for Epidemiological Studies Depression Scale
GBM: gradient boosting machine
LASSO: least absolute shrinkage and selection operator
ML: machine learning
MOST: Multicenter Osteoarthritis Study
OA: osteoarthritis
OAI: Osteoarthritis Initiative
PASE: Physical Activity Scale for the Elderly
SF-12: 12-item Short Form Health Survey
WOMAC: Western Ontario and McMaster Universities Osteoarthritis Index
